# What Anæsthetics Shall We Use

**Published:** 1878-04

**Authors:** Julian J. Chisholm

**Affiliations:** Professor of Eye and Ear Diseases, University of Maryland, and Surgeon in charge of the Baltimore Eye and Ear Institute.


### AMERICAN JOURNAL OF DENTAL SCIENCE.

Vol. XI. THIRD SERIES—APRIL, 1878. No. 12.

### ARTICLE I.

### *What Anæsthetic Shall We Use?*—Continued.

BY JULIAN J. CHISHOLM, M. D.

Professor of Eye and Ear Diseases, University of Maryland, and Surgeon in charge of the Baltimore Eye and Ear Institute.

*Anæsthetics are among the most powerful drugs of the Pharmacopœia, and must always be administered with caution.* "To bring a living being to that borderland in which life in many respects so simulates death should at no time be a fool's occupation." It is a condition which the strongest hearted patient can not face without some uneasiness, and many accept with great alarm. The administration of anæsthetics should therefore be only entrusted to skilled hands, who, knowing what they have to do, will give all of their attention to the serious matter which should absorb them. Hence it is better to have as an administrator of anæsthetics a physician who takes no interest in surgical operations, and who will not have his attention for even a moment diverted from the face and pulse of his patient.

From my own experience and observation, I can readily see how there can be *seven causes for death* during the administration of anæsthetics, even when the chemically pure drugs are used, the patient being in the recumbent posture, with loose clothing, and in a proper position for its inhalation. Etherization in the sitting posture is dangerous, and should never be undertaken. May not the perfect safety with which chloroform is used in obstetrical practice depend much upon the empty condition of the stomach, the loose clothing, *and the horizontal decubitus*, which are considered the best preparation for safe inhalation, accompanied, as it always is in labor cases, with no fear of, but a longing for, the anæsthetic.

*In the first place*, I can understand how a cloth saturated with chloroform, and covering up the face of a patient so as to exclude air, may necessitate the inhalation of so saturated an atmosphere as to cause excessive irritation of the air-passages. This may act injuriously upon the respiration and circulation. Should a fatal result occur under this condition of faulty administration, we would not call this a death from chloroform under careful use, but its careless abuse; a result that could not occur if common precautions are taken, such precautions as every physician is expected to use who administers any of the efficient drugs of the materia medica. Should a physician intending to administer hypodermically a few minims of Magendie's Solution from a full syringe carelessly inject the entire contents under the skin, no one would attribute the blame to the morphia, but to the hand that worked the syringe. In this case life is sacrificed by carelessness in the use of a remedy known to be potent, and therefore one to be used with caution.

*A second cause* of death may readily occur with the early administration of both ether and chloroform. In many cases the local action of the inhaled vapor so irritates the throat as to cause contraction in the tongue muscles and a closure of the laryngeal opening by the close approach of the arytenoid cartilages and the overlapping of the epiglottis. This condition is evinced by stertorous breathing, change of countenance, discoloration of face, impeded thoracic movements, and vain attempts at thoracic expansion. The patient is threatened with asphyxia by an involuntary but successful effort at partially swallowing his tongue. Should he be allowed to remain in this condition, death will as surely take place as when one is seized by the throat and fatally strangled. If the mouth be opened, tongue caught by the tongue-forceps and drawn forcibly outwards, to one side, so as to elevate and advance the epiglottis and separate the arytenoid cartilages, thereby re-establishing the laryngeal opening, a loud, sonorous inspiration is immediately heard, air pours into the lungs, and the ominous cloud upon the face at once passes away. Should there be no forceps at hand, and the drawing out of the tongue be omitted, we would have, under these conditions, another glaring instance of death from the maladministration of the anæsthetic. A surgeon who is not prepared to protect his patient from this accident, while inhaling an anæsthetic, would have as good reasons for escaping censure as he who, after an amputation, found that he had omitted to procnre the instruments necessary for securing the arteries, and hence allowed the patient to die from hæmorrhage.

*A third cause* of danger in the inhalation of anæsthetics resides in the inexperience of the administrator or in his inattention. I have already said that the physician who administers an anæsthetic has full occupation in the part assigned to him, and should not permit his attention to be diverted to the surgical work performed by another, and for which he is preparing the patient. I have seen instances in which the administrator of the anæsthetic, often a student of medicine, in his eagerness to follow the several steps of the operation, had become so oblivious to his responsible trust as to allow the thickly folded cloth to compress firmly both nose and mouth of the patient, who with asphyxia imminent, was too insensible and powerless to push away the obstacle and escape the threatened suffocation. Had the patient a feather pillow pressed over the face he could not be more surely suffocated than by this thickly folded towel. Many a time in doing general surgical work have I pulled the cloth from the face to the immediate relief of the respiration. In opthalmic surgery, to which my work is now exclusively confined, this accident can not occur, as the face is constantly under my observation. Had the careless administrator been permitted to smother his patient we would have had a death announced from chloroform, while a towel which had never known chloroform if applied in a similar way would have done equally efficient work. Another clear case of careless administration.

Still a *fourth cause* of death will be wrongfully attributed to the anæsthetic should it occur doing the act of vomiting. If the patient be allowed to remain upon his back during emesis, and be not rolled over upon his side or face, some of the contents of the stomach will pass downwards from the pharynx into the trachea and cause death by suffocation. Autopsies are not often thoroughly made after supposed death by anæsthetics. Usually the brain, the heart and the kidneys are examined, and possibly the stomach, liver and bladder, but not the throat and the larynx. But in one recently reported death during chloroform inhalation, in which a patient suddenly expired immediately after the act of vomiting, the larynv and trachea were found packed with food ejected from the stomach. In another recent case at the University College Hospital, London, of death following vomiting under Ether during an operation for strangulated hernia, the autopsy exhibited stercoraceous matter in the trachea and right bronchus. Again, a case of death at the hands of the careless administrator in his bad manipulation of the patient, and certainly not from the anæsthetic.

*The fifth cause* of death from both chloroform and ether, probably the most common of all, has been more active of late years, being rendered so by the unsettling of the former confidence which surgeons have had in the safety of anæsthetics. In their present timidity surgeons do not now push the inhalation to the degree of suspending the functions of such parts of the cerebro-spinal system as preside over the emotional, sensational, motor and reflex acts; *the only condition recognized as one of perfect safety in chloroform and ether anæsthesia.* I refer to that condition in which peripheral irritation can no longer be transmitted through the cord to the brain, and then back, by the vagus and pneumogastric nerves, to the cardiac ganglia. Any condition short of this stage of temporary suspension of reflex agencies leaves the heart exposed to those serious inroads from peripheral irritation through which its movements may be suddenly and permanently arrested. Such fatal results are identical with instances of nervous shock, so familiar to operators in former times, and then deemed a sufficient explanation of death under the circumstances. In this way can be satisfactorily classified the many deaths under anæsthetics for trivial operations, as tooth drawing, opening of abscesses, etc., when only enough of the agent was inhaled in the sitting posture to partially stupefy, but not to protect against reflex accidents from emotional or peripheral excitement.

In this class are placed those many fatal cases during the more serious operations, in which the timidity or anxiety of the surgeon, with unsettled confidence in the article he is using, induces him to *arrest the inhalation before the period of safety has been reached.* He commences the cutting operation with the vital organs all exposed to injurious reflex peripheral irritation, and under the cloak of the anæsthetic, without its protection, invites disaster. Hence it is that an operator, who has once been frightened by anæsthetics, continues to have accidents which do not occur to others who, never having seen trouble, administer the drug boldly. Timidity here must be classified with ignorance, both being dangerous negative qualifications for successful surgery. When deaths occur under these circumstances, the fatal result is not to be attributed to the anæsthetic, but, on the contrary *to the want of it;* clearly a defective administration, induced by unwarrantable and unworthy fright on the part of the operator.

Under this heading I would also class the deaths said to be occasioned by heart diseases under the inhalation of chloroform. Richardson, of London, after a critical examination of the various diseased conditions of the body, said to be hostile to the administration of chloroform, and especially after maturely considering the many varieties of heart disease, including valvular growths, vascular contractions, cardiac, hypertrophies, stenosis of heart orifices, and in fact the entire list of heart troubles, sums up his experimental observations with the following remarks: "On the whole, the only diseased condition which I could give as a warning to practitioners from exceptional danger in the administration of chloroform is the diagnostic of a dilated and weak right heart." And then states that in one such case he had forewarned the surgeon, who then gave chloroform and had a fatal result. But what guarantee have we that the patient with such a heart would not have succumbed to the operation itself? These heart conditions are so prone to syncope that anæsthetics are needed during painful operations to prevent the fatal emotional shock. In such cases we recognize the necessity for a cardiac stimulus, whiskey, which may have prevented the fatal issue.

*Diseased conditions of the heart, regardless of kind,* may make this important organ peculiarly susceptible to syncopetic influences when reflex action has full sway; hence we find violent emotional excitement a fruitful cause for mortality in the subject of heart disease. Many such persons having to undergo painful surgical operations in former times, before the introduction of chloroform, suddenly collapsed with the first incision; and they *still die as of old when they are not properly protected by complete anæsthesia.* Should chloroform be freely given to patients with heart disease, regardless of kind, who must submit to painful operations for the cure of some surgical affection, by its liberal use they are put in a condition of safety against all emotional and reflex annoyances, without which they could not escape trouble.

*I look upon chloroform as the strong bridge which will conduct patients suffering from serious heart disease safely through serious operations.* As a surgeon in large opthalmic practice, I frequently am compelled to perform the most delicate and painful operations upon the eyes of timid patients suffering from heart disease in its various forms. Cataracts occurring usually at an advanced age, most frequently between 60 and 80 years of age, are often associated with organic diseases of the heart in patients enfeebled by senility. I never refuse to give such patients chloroform; on the contrary, I urge its use. The only difference that I make in such cases over other patients is, if possible, by exercising more care in establishing the safe stage of complete anæsthesia through the liberal use of the drug. From the standpoint of my own personal experience, I know of no organic lesion which contraindicates the careful and thorough administration of chloroform.

The *sixth cause* of death during the administration of ether or chloroform is from excessive administration. No one can doubt for a moment that chloroform and ether possess toxic action, and that in common with all other active agents in medicine, the danger is dependent upon the size of the dose used; also that the dose of an anæsthetic can be made large enough to kill by enfeebling and finally paralyzing the nerve centres from which the heart and lungs draw their inspiration. This class of remedies are clearly cumulative, and when enough has been inhaled to cause the suspension of voluntary motion, sensation and reflex action, if their administration be continued, instead of being suspended, an amount can be concentrated in the circulation quite sufficient to stop respiration and the heart's action.

As I have said before, there is a broad gulf between that degree of anæsthesia which only suspends so much of cerebral action as still allows full play to the vital organs—a perfectly safe condition for surgical operations—and that fatal overdose of the anæsthetic from which there is a suppression of the cardiac centres of innervation. Surgeons often employ ignorant assistants to administer the anæsthetic who conceive it to be their duty to keep the saturated cloth to the nose of the patient, and they know no stopping point. They are told to give the ether, and they show neither judgment nor discretion. Unless watched by the surgeon, whose attention should be concentrated elsewhere, they go on applying the vapor in spite of complete narcotism. Fortunately, the elimination of the gas from the blood through the lungs is so rapid that by removing the towel a very few expirations will reduce the amount in the system to a safe standard and dissipate the threatened danger. When through this overdose the vital functions are suspended, the killing should be put to its proper cause—an overdose through ignorance; another clear case of mal-administration.

The *seventh, the only legitimate and rarest of all causes of death from anœsthetics,* now faces us. It is that unknown condition called idiosyncrasy, in which anæsthetics show themselves poisons of extreme activity. The patients who carry about with them this innate fatality exhibit it by no recognized signs. When such persons die from the toxic inhalation, the autopsy reveals absolutely nothing to indicate the destructive effects of the poison.

The effects of anæsthetics, says Claude Bernard, depend on the immediate influences exerted by the drug upon the sensitive elements of the nerve centres, in virtue of which their properties are temporarily suspended. In cases of idiosyncrasy, these functions, so essential to life, become permanently suppressed, and yet, with the death of the individual, the changes in these nerve elements are of such a nature that so far they have altogether escaped detection by pathological investigators.

We call the practice of medicine empirical, because every dose of medicine we administer to a patient for the first time is more or less an experiment. We cannot in advance say that because a drug is expected to act in a previously established way it will do so in the case before us. The every-day experience of physicians teaches them not to be surprised if now and then they should obtain from drugs diametrically opposite results from those looked for, and very often intensity of action not at all commensurate with the very small dose administered. I have known opium to produce intense pain; it is the common remedy to allay it. I have known Dover's powder to purge violently, a similar effect to be repeatedly produced from a few grains of quinine or a single dose of bromide of potassium. I have known one-half grain of iod. pot. to cause a most distressing coryza, and a fragment of a grain of mercury to excite the most profuse ptyalism. On one occasion in an old lady under my care, one single drop of Fleming's tincture of aconite, brought on prostration that nearly proved fatal. How often a single moderate dose of the narcotics in constant use has put the patient under the sod, the grave alone can tell. To the world without the death is always attributed to the disease for which the dose has been judiciously prescribed. The administration of the remedy was based upon the great good that it had accomplished in controlling these very symptoms in thousands of cases. The idiosyncrasy of the individual in these special instances was the immediate cause of the fatal issue; a condition which could not have been foreseen, and therefore no precautions could have guarded the physician against it. In these cases, which can not be very rare, from the variety and number of potent drugs used by practitioners, the fatality following the dose administered is often not recognized, even by the medical attendant. The patient died when the symptoms of the disease had not indicated so serious or so sudden a result, but the reason why, most physicians do not consider.

From the exclusive use of any one potent remedy the idiosyncrasy must be rare, so that in taking one, say opium, a powerful drug most extensively used by every physician, and applicable to most of the diseases to which the human subject is liable, peculiarities in constitution, exhibiting dangerous symptoms from comparatively small doses, are only now and then met with. When we contrast as to frequency cases requiring serious surgical operations, against the many little and great disturbances of the various organs of the living economy brought to the notice of the physician and requiring the use of opium, we find the surgical cases in the ratio of scarcely one to a thousand. Now take such a remedy as chloroform, only used by surgeons in these serious surgical cases, and hunt up idiosyncracies for this drug. Their occurrence must be so very rare that a surgeon of very large experience is not likely to see more than one fatal case in a long life devoted to surgical practice; and a great many surgeons of very large experience have never met with one. Syme, whose surgical career in Edinburgh is known to every one in the profession, was so uniformly successful with anæsthetics, never having lost a patient from the inhalation, that he adopted this axiom, "Show me a case for operation, and I will show you a case for chloroform." At the Edinburgh Infirmary, during a period of 28 years from the introduction of chloroform into surgical practice to the present time, only two deaths have been attributed to chloroform, which, according to Ker, is one death in 36,500 administrations. Grant, in his admirable Treatise on Surgery, says: "I have seen chloroform given in some thousands of cases during upwards of twenty years, both in hospital and private practice, without a single death, or even an approach to a fatal termination." Elser, of Strasburg, had used chloroform 16,000 times, and had never seen a fatal case. Kidd, of London, had seen it administered upwards of 10,000 times, and had seen no fatal case, either in his own practice or that of his friends. The French surgeons in the Crimea reported 30,000 cases of chloroform administered and not one fatal issue. In the English army in the Crimea chloroform was administered 12,000 times with one single death reported as attributed to it. In the Confederate service chloroform was exclusively used in a great many thousand operations without a death, as far as I am aware of, or have been able to ascertain after diligent inquiry among leading surgeons of the army. Surgeon McGuire, of Jackson's corps, reported 18,000 administrations without one death. Richardson had seen it used in the London hospitals 15,000 times before he met with the first fatal case. Billroth, of Vienna, had administered chloroform 12,500 times before he met with his first accident. Clover has recorded 3,000 administrations without a single death. Erichsen has only witnessed one single death under chloroform in 25 years at University Hospital. No official statistics, that I am aware of, have been published of the uses of chloroform in the Federal army, nor in the recent wars of the French, German and Austrain empires. Dr. J. Mason Warren, in 1867, published his "Surgical Observations, with Cases and Operations," in which he mentions that in the Federal army chloroform was almost exclusively used in field operations. "The returns indicate that it was administered in no less than 80,000 cases. In 7 cases fatal results had been ascribed with apparent fairness to its use," a proportion of 1 death in 11,428 administrations. Enough has been already said, however, to prove that under careful administration, deaths from chloroform must be among the rarest of accidents—so very rare that it should not be seriously considered.

To the testimony above I will add my own individual experience. I have been practicing surgery twenty-five years, and have used chloroform largely during that entire period in private and hospital practice, in the army as well as in civil life, and have administered it to the extent of fully 6,000 cases. For some years I have administered it on an average of at least once every day. I have given it to the very young and to the very old; to the very strong as well as to the very weak; to the healthy as well as to the extremely diseased, regardless of the organ in which the trouble may be located. I have seen patients thoroughly anæsthetized by a half drachm of chloroform, and I have used an entire pound upon an individual before I could secure full narcotism, and I have had occasion to keep up the anæsthesia as long as three hours at a time. I have accepted Syme's axiom, and given chloroform to every one, regardless of visceral complications, who has applied to me for a serious surgical operation, and I have yet to see the first death, either in my own practice or that of my friends. Now let us sum up the evidence which I have collected, and here we find an array of *over* 250,000 *administrations of chloroform with* 12 *deaths*, even attributing them all to idiosyncrasy, which calls for a most unbounded charity, and we only have 1 death in 20,000 cases. Can any stronger proof of the excessive rarity of the fatal idiosyncrasy in chloroform be needed?

With ether, although we have no statistical records, I believe that deaths during its careful and full administration are equally rare; and that in America, where it was discovered and has been most used, especially by the Boston surgeons and some in New York (for its administration seems to be chiefly confined to the Northern cities,) we may find surgeons like those above mentioned who have records of thousands of cases without a single fatal issue. And yet, in proof that neither anæsthetic is absolutely safe, deaths, however rare they may be, do sometimes occur during the administration of both ether and chloroform, even when the purest article has been used and every care bestowed in the inhalation. Some surgeons seem to have been unlucky enough to have had a great deal more trouble with anæsthetics than should have fallen to the share of one administrator. I, for one, do not believe in lucky and unlucky surgeons. I believe, with Napoleon, that luck usually accompanies the heavy and best organized battalions.

Against the fatality of idiosyncrasies we can hardly guard, and yet something even here might be done. Three or four times in my own experience I have had cases in which I at the time thought that the anæsthetic which I was administering was badly borne. Once while giving chloroform I noticed a sudden and unusual pallor. I stopped the administration, and the patient, by breathing pure air, soon assumed a natural appearance. I resumed the chloroform with similar results. I then exchanged it for ether, and had no further appearance of these symptons. What might have occured had I continued the chloroform, I am unable to say, possibly nothing but the most satisfactory anæsthesia. It might have been a groundless fright, still I am willing to call it an idiosyncrasy. In a second case, a young girl of seventeen, to whom I had given no stimulus, I thought that the pulse was rapidly enfeebled by the chloroform inhalation, and I exchanged the anæsthetic for ether. These cases occured some years ago. More recently, since the sulphuric ether has been urged as the safer anæsthetic, I, with no reason for doing so beyond popular clamor, have used it very freely. In one or two instances I thought it was badly borne, causing intense congestion of the head or excessive irritation of the throat. In these cases I stopped the ether and administered chloroform, with, as I conceived, marked relief. My fears here again might have been altogether groundless, as in the chloroform cases before mentioned.

I constantly see cases which excite the most anxious solicitude on the part of the timid and inexperienced operators —a marked enfeebling of the pulse, feeble respiration, pallor of the face, and relaxation of the skin with perspiration pouring out upon the surface. Experience has taught me that this relaxed condition, which so many are terribly alarmed about, is only the precursor of vomiting, and is the signal that I must prepare the patient for emesis. This condition, so constantly met with by the every-day administrator of chloroform, has so frightened many an inexperienced or timid operator as to make him believe that he had come within an ace of having a fatal case of inhalation on his hands.

When one uses chloroform or ether in the ways as explained, he might confidently expect no trouble. Should he believe that chloroform always weakens the hearts action, he puts in anticipation the best of cardiac stimulants, a drink of whiskey, in the stomach of the patient, where it is ready for use if wanted, and can do no harm if it is not required. I attribute the uniform success of chloroform inhalation, in the hurry and confusion of battle field surgery, to this invaluable combination of whiskey with chloroform, and this in the face of the fact that as the Government purchases from the lowest bidder, army supplies are never of the best, and in times of war, with heavy demands, army medicine supplies are very far from being chemically pure.

Suppose, however, that from the tediousness of the operation or otherwise there should be a very marked enfeebling of the heart's action, the course to be pursued is very simple. I believe that it is now conceded that chloroform produces anæmia of the brain, and that the various phenomena observed during the administration is in a measure caused by the diminishing blood supply to the various nerve centres. Every one has observed the suddenness with which the anæsthetic effect is diminished with the act of vomiting, with its accompanying congestion of the head. Nelaton took this ground, and has given to the surgical world that admirable mode of restoring vigor to the heart by hanging the patient up by the feet, so as to allow gravity to supply the needful stimulus to the brain. When there is no longer the anæsthetic to the nose of the patient, as every expiration is getting rid of a certain amount of chloroform vapor from the circulation, fresh air would naturally suggest itself as a substance to be freely admitted. Should the respiration cease while the heart still acts, however feebly, artificial respiration should be at once instituted and continued either until the heart altogether ceases to beat, or until resuscitation is fully established. If the respiration and heart's action be detected, however feebly, ample experience shows that fresh air and an inclined position with head downwards is all that is wanted for a re-establishment of the vital functions; and that death which ought not to occur under this condition may often with truth be attributed to the too much manipulation of the frightened attendants. Electricity may help the respiratory effort should it be *properly applied with proper apparatus at hand;* but according to Richardson's experience and observation it is most frequently the name of electricity application only, and in by very far the majority of cases it does more harm than good. For if not scientifically applied it will insure the killing by permanently stopping both heart and lung action.

In those most rare but truly unfortunate cases in which the heart stops beating and remains so for only one single minute, the patient is dead absolutely, and nothing that the surgeon can do will restore him to life. The surgeon, unwilling to acknowledge his utter helplessness, keeps up much doing of many things for many minutes or hours, but all to no avail. These fatal cases should be only the very rare ones of idiosyncrasy which we may hear much of, but may never see, and yet they may occur to the most careful. The majority of deaths ascribed to chloroform properly should be attributed to mal-administration; *a fruitful source of trouble being that timidity of surgeons which will not allow them to safely anæsthetize patients,* but drives them to operate before a sufficient amount of the anæsthetic is administered to protest against the dangers of reflex action. I truly believe that a great many more cases of death under chloroform are to be attributed to the want of it than to an overdose, which comes only next in rarity to deaths by idiosyncrasy; and that the timid surgeon and not the good chloroform swells the mortuary list.

When persons suddenly die on the street or at their homes, the coroner is ever ready with his convenient verdict of heart disease, when most frequently the heart is a perfectly healthy organ, and has been altogether innocently accused. If in any case an anæsthetic has been used and a fatal accident occurs, death is immediately ascribed to the inhalation, when in reality it is due to other causes altogether extraneous to the administration. Notwithstanding this gross error in diagnosis, the reported death has its disturbing influence upon the profession; and when frequently reproduced in the daily papers will frighten the masses.

As chloroform has been up to within a few years the anæsthetic in nearly exclusive use, very naturally a great many more deaths have been attributed to it than to the much less used ether. With its increased use, deaths from ether are now accumulating and could rigorous statistical accounts be obtained, it would be found that ether, in proportion to the comparably small number of inhalations, would relatively exhibit as many deaths as chloroform. Up to within a few years surgeons at large have had every confidence in chloroform, and the language is not strong enough to express their unbounded admiration. Recently this confidence has been disturbed by the much talked-of toxic effects of chloroform, which has frightened the public and excited the timid in the profession until many have imbibed the infection, not knowing why, and have taken to ether, a substitute of limited use, which has not had the opportunity of having as many deaths attributed to its administration. I also three years since, under the pressure of public opinion, or rather the timidity of patients who expressed a preference for it, took up ether and gave it largely. For a short time I used nothing else, but its administration proved unsatisfactory, on account of the great distress occasioned by its forced inhalation in a concentrated form, its offensive odor, the large amount required, the excessive nausea induced, and the irritable cough often excited. As we have all so often done with new remedies, relinquish them in favor of the older ones which had previously been our reliance, so I found myself getting back to chloroform, which I now exclusively and daily use with all the confidence that so useful and safe an agent ought to secure.

*Believing, as I do, that both ether and chloroform can kill, when carelessly, indifferently or excessively administered*—believing also that either of them will kill when the idiosyncrasy is met with in which its usual benign effects become toxic, and that these two remedies will do so in equal ratio to the number of times in which they are inhaled I naturally confide in the one which experience has taught me to be equally safe, more agreeable, less nauseating, and more efficient. My acquaintance with chloroform has been of the most satisfactory kind. I have seen it administered at least six thousand times, and I have never seen trouble from it. Its effects have been uniformly good.

Since my attention has been turned to the decided advantages of chloroform over the less efficient sulphuric ether, I have often asked surgeons from a distance, with whom I may have been casually thrown, what anæsthetic they use. I find many say chloroform exclusively, from which they had never had an accident, and in which they have unbounded confidence. Others tell me that they administer ether; not that they had ever had trouble with chloroform, but that they had been made somewhat timid by reading reports of fatal cases. They at the same time acknowledge that they do not obtain that satisfactory anæsthesia as they formerly did under chloroform; and that even now, when they find that their patient is not properly influenced with the ether, they pour on the chloroform, feeling that they can look confidently forward to a speedy and thorough anæsthesia.

Believing, as I do, that ether and chloroform will not prove dangerous if a pure drug is selected and carefully administered, except in those extremely rare cases of idiosyncrasy, when both will prove toxic in like proportion, then in the comfort of the administration, both to the patient and to the surgeon, ether, in my opinion, is not to be compared to chloroform as a general anæsthetic, and can never take the place of the latter. The wave of professional opinion must move back toward a returning confidence in the safety of chloroform, which many surgeons who administer it continually have never had shaken.

There is no remedy really good in medicine which is not capable of mischief. More persons are killed every year from the abuses of opium than have been attributed to chloroform from its discovery even to the present time. Deaths from opium in England alone number three hundred per year. How many hundreds are to be added to this occurring under the careful administration of skilled physicians, we can easily conjecture. Yet who proposes to substitute for this king of drugs any of the milder narcotics? We are ready to accept the comparatively small fatality for the immense good it accomplishes. How often during the summer do we find in the daily papers reports of healthy persons dying suddenty from the too liberal potation of ice-water, yet who would listen with any patience to the so-called philanthropist who would wage war against this universal beverage? It has been stated that more persons are killed by slipping on fragments of orangepeel in the streets of London than from the inhalation of chloroform. No one suggests the destruction of orange groves on this account. No one finds fault with the laws of gravitation because persons continue to fall down and break their legs or necks. It is unfortunate for the individual, but the great laws of Nature must ever go on, and the great designs of the Creator enjoyed with wonder and astonishment. Shall we give up the use of horses or steam because accidents happen from both? Would we have all the rivers dried up because now and then a drowned man is fished up out of them? The great discoveries are for the good of the many —not for the few. You can not avoid every danger and supply every good.

With anæsthetics the little risk, infinitesimally small, is immensely counterbalanced by the protection from the very many dangers during operations, so well known and so often experienced when operations were performed without them. Chloroform, when judiciously used, is one of the safest active remedies of the materia medica, supplying nearly every good and avoiding nearly every danger.

"*The greatest gift of God to man through natural science is chloroform.*"

